# The Mechanism of Stimulating and Mobilizing the Immune System Enhancing the Anti-Tumor Immunity

**DOI:** 10.3389/fimmu.2021.682435

**Published:** 2021-06-10

**Authors:** Zhengguo Wu, Shang Li, Xiao Zhu

**Affiliations:** ^1^ Department of Thoracic Surgery, Yantian District People’s Hospital, Shenzhen, China; ^2^ Guangdong Key Laboratory for Research and Development of Natural Drugs, Guangdong Medical University, Zhanjiang, China; ^3^ Central Laboratory, The First Affiliated Hospital of Wannan Medical College (Yijishan Hospital of Wannan Medical College), Wuhu, China; ^4^ Guangdong Provincial Key Laboratory of Systems Biology and Synthetic Biology for Urogenital Tumors, Department of Urology, The First Affiliated Hospital of Shenzhen University, Shenzhen Second People’s Hospital (Shenzhen Institute of Translational Medicine), Shenzhen, China; ^5^ First Affiliated Hospital, Bengbu Medical College, Bengbu, China

**Keywords:** anti-tumor immunity, CAR-T, checkpoint inhibitors, immune system, immunotherapy

## Abstract

Cancer immunotherapy is a kind of therapy that can control and eliminate tumors by restarting and maintaining the tumor-immune cycle and restoring the body’s normal anti-tumor immune response. Although immunotherapy has great potential, it is currently only applicable to patients with certain types of tumors, such as melanoma, lung cancer, and cancer with high mutation load and microsatellite instability, and even in these types of tumors, immunotherapy is not effective for all patients. In order to enhance the effectiveness of tumor immunotherapy, this article reviews the research progress of tumor microenvironment immunotherapy, and studies the mechanism of stimulating and mobilizing immune system to enhance anti-tumor immunity. In this review, we focused on immunotherapy against tumor microenvironment (TME) and discussed the important research progress. TME is the environment for the survival and development of tumor cells, which is composed of cell components and non-cell components; immunotherapy for TME by stimulating or mobilizing the immune system of the body, enhancing the anti-tumor immunity. The checkpoint inhibitors can effectively block the inhibitory immunoregulation, indirectly strengthen the anti-tumor immune response and improve the effect of immunotherapy. We also found the checkpoint inhibitors have brought great changes to the treatment model of advanced tumors, but the clinical treatment results show great individual differences. Based on the close attention to the future development trend of immunotherapy, this study summarized the latest progress of immunotherapy and pointed out a new direction. To study the mechanism of stimulating and mobilizing the immune system to enhance anti-tumor immunity can provide new opportunities for cancer treatment, expand the clinical application scope and effective population of cancer immunotherapy, and improve the survival rate of cancer patients.

## Introduction

**Figure 1:**
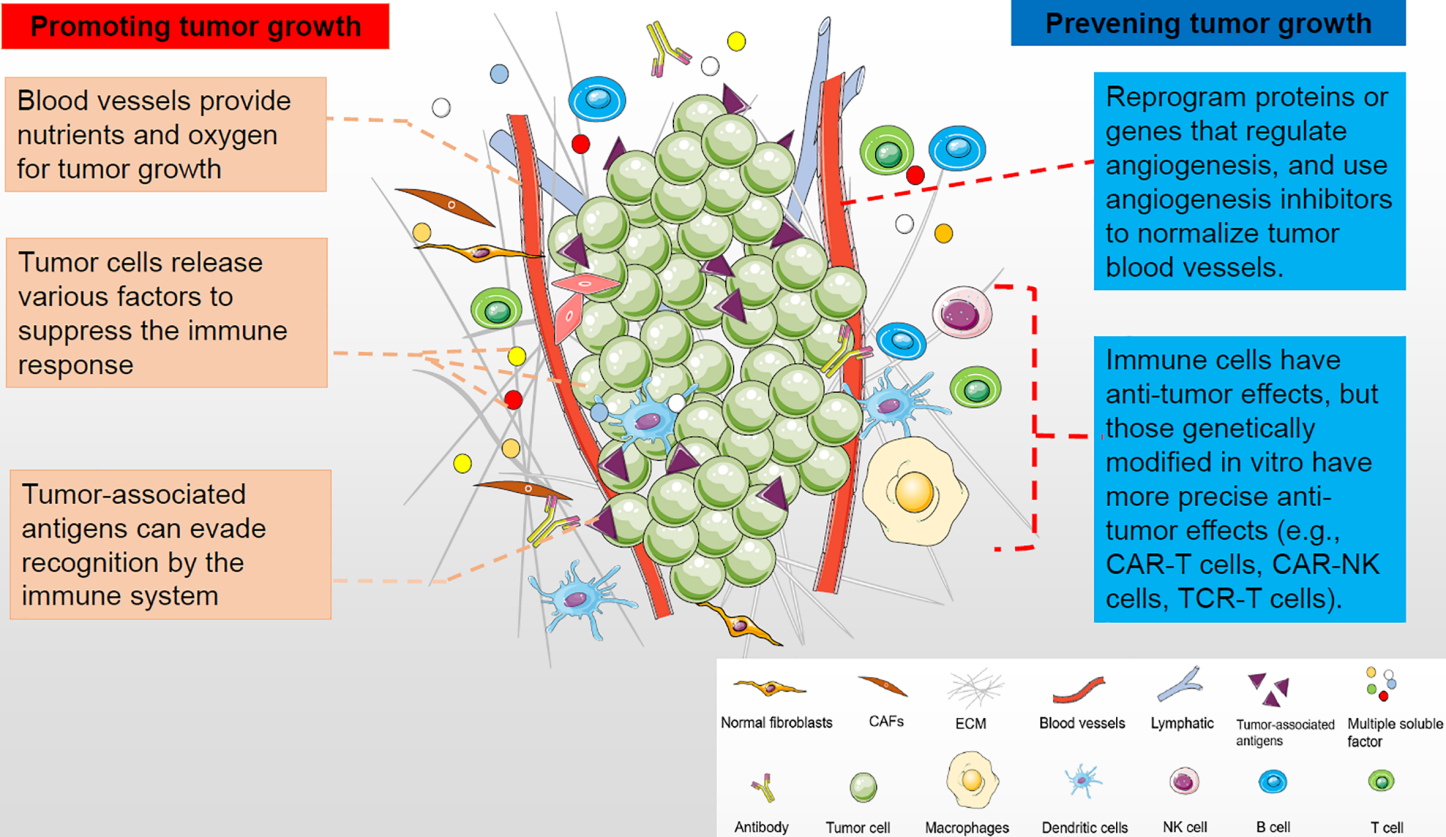
The tumor microenvironment is composed of cellular and non-cellular components that support tumor growth. Tumor and its microenvironment interact and promote each other through angiogenesis and immunosuppression. Therefore, targeting tumor microenvironment in anti-tumor therapy can make greater progress, such as inhibiting tumor angiogenesis and tumor immunity.

Tumor microenvironment (TME) is a complex environment in which tumor cells depend for survival and development. The tumor microenvironment is composed of cellular components and non-cellular components (1). The cell components include tumor cells, inflammatory cells, immune cells, mesenchymal stem cells, endothelial cells, and tumor-related fibroblasts. Non-cellular components, including cytokines and chemokines, constitute a complex tumor microenvironment. These cellular and non-cellular components work together to support tumor growth (**Figure 1**). Tumor and its microenvironment interact and promote each other through angiogenesis, immunosuppression and other means (2). Therefore, the influence of tumor microenvironment on treatment must be considered when developing tumor treatment methods, or targeted tumor microenvironment treatment methods should be adopted. In the past, researchers have focused on targeting cancer cells themselves, such as traditional chemoradiotherapy and targeted therapies that target the tumor itself. In recent years, the treatment of tumor against tumor microenvironment has become a hot spot of tumor treatment. Studies have found that this kind of treatment can often bring better results. Anti-angiogenesis therapy and immunotherapy are the mainstream treatment methods for tumor microenvironment at present (**Figure 1**). The aim of tumor immunology therapy is to stimulate or mobilize the body’s immune system and enhance tumor microenvironment anti-tumor immunity, so as to control and kill tumor cells (3). There are many methods of tumor immunological therapy (4) (5), including tumor vaccine (6), immune-guided therapy, cell adoptive immunotherapy (7), cytokine therapy, gene therapy (8) and comprehensive therapy. Immunotherapy of tumor, whether a single drug or combination therapy, has shown clinical efficacy in many kinds of cancer, so it has attracted more and more attention (3, 9). Although immunotherapy has great potential, it is currently only applicable to patients with certain types of tumors, such as melanoma, lung cancer, and cancer with high mutation load and microsatellite instability, and even in these types of tumors, immunotherapy is not effective for all patients. In addition, the skin toxicity, gastrointestinal toxicity and other side effects caused by immunotherapy of tumor should not be ignored (10). This article reviews the mechanism of immune resistance, principle of immunotherapy for cancer, the application of new immunotherapy for cancer and immunotherapy in different tumors. It also introduces the new strategies to overcome the resistance of immunotherapy for cancer, the methods to improve the curative effect of immunotherapy and to alleviate side effects, as well as the screening, treatment and effectiveness evaluation of beneficiaries before immunotherapy.

## Mechanisms of Immune Resistance

Standard immunotherapy has not been as effective as expected in the treatment of aggressive and advanced cancers. This is due to decreased immunogenicity and increased immune tolerance. When the immune “hot” tumor becomes “cold” or immunosuppressed, the tumor recurs (11, 12). Lack of understanding of these mechanisms may hinder the development and clinical application of tumor immunotherapy.

### Tumor Cell Heterogeneity

Tumor heterogeneity refers to the presence of cells with many different genotypes or subtypes within the same tumor (13–15). Therefore, the same kind of tumor in different individuals can show different therapeutic effects and prognosis, and even the same body tumor cells have different characteristics and differences. Subclones usually survive treatment and lay quiescent. When selective pressure is removed, the cells can produce signaling factors that increase the tumorigenicity and growth ability of tumor cells, a process known as subcloning synergy. The influence of drug-resistant subclones is the basis of the limited efficacy of current immunotherapy (16).

### Major Histocompatibility Complex

The loss of MHC Class I and Class II molecules has been shown to be associated with tumor progression. Human MHC or Human Leukocyte Antigen 1 (HLA-I) molecules are encoded by a series of polymorphic genes with multiple alleles or haplotypes. The most common method for these allele alterations is loss of heterozygosity in the coding region of HLA and 2-microglobulin (an essential element of the HLA-I complex), resulting in loss of HLA-I haplotypes, and loss of chromosome 15 (7, 17). Tumor cells also have a tendency to evade NK cell detection by regulating the expression of MHC class I-like NKG2D ligands.

### Anti-Apoptotic Mechanisms

Tumor cells use a complex network of hyperactivated signaling pathways to protect cells from apoptosis and ensure their continued survival and proliferation. Common signaling pathways are Ras/Braf/Mek/MAPK pathway and PI3K/Akt pathway. Activation of MAPK pathway can promote the expression of tumor immune editing, immunosuppressive cytokines and checkpoint markers, and reduce the infiltration of CTL (18, 19). The hyperactivated PI3K/Akt pathway has these functions after the loss of its physiological inhibitor, the phosphatase and tensin homolog (PTEN). Abnormal PI3K/Akt signaling pathway inhibits cell apoptosis by up-regulating the expression of Bcl-2 and reducing the level of apoptotic regulators.

### Immune Checkpoint Ligands

The coinhibitory checkpoint ligand on the APC interacts with the receptor on the lymphocyte to prevent self-reactivity and maintain peripheral tolerance. When a programmed death receptor of T cell encounters these checkpoint ligands and is unable to fully respond to its target, the T cell experiences impotence or failure (the “off switch”) (20, 21). Although a series of features of this ligand are normal tissue, tumor cells are known to have the ability to inhibit tumor-specific t cell function. PD-L1 and CTLA-4 are the most studied checkpoint ligands (6, 22).

## New Approaches to Tumor Immunity

### Cancer Vaccines

Tumor vaccines amplify tumor-specific T cell responses by generating active immunity by identifying tumor-associated antigens. The active components of cancer vaccines consist of four key components described below: tumor antigens (6), preparations, immune adjuvants, and delivery vectors (23). There are two types of cancer vaccines. One is used to prevent cancer. The other, which is used to treat cancer, is emerging immunotherapy. It stimulates the body’s immune system to fight, killing cancer cells and preventing them from spreading and returning.

New York esophageal squamous cell 1 (NY-ESO-1), a kind of cancer/testis antigen (CTA), is a cancer-associated protein found in many invasive tumors (24). It is widely expressed in breast cancer, bladder cancer, prostate cancer, melanoma, NSCLC, hepatocellular carcinoma, ovarian cancer and other cancers, with a range of 20-80%. It is one of the most immunogenic antigens in the body, so it is considered as an ideal target antigen for tumor immunotherapy. NY-ESO-1 cancer vaccine is made with NY-ESO-1 as the target antigen. DC cells ingested cancer-specific antigens composed of NY-ESO-1 derived peptides and presented them to the tumor microenvironment. DC vaccines containing NY-ESO-1 peptide directly stimulate T cells to fight tumors (25). Racotumomab is a murine gamma-type anti-idiotype monoclonal antibody that specifically induces an antibody response against Neu-glycolyl GM3 ganglioside. The best clinically active heat shock protein for advanced NSCLC patients ACTS as an intracellular chaperone, binding and presenting tumor antigens on specialized APC *via* MHCI and II molecules, leading to activation of anti-tumor T cells. Racotumomab has been shown to be a maintenance therapy for advanced non-small cell lung cancer (26). The tumor antigen of the CryoVax vaccine comes from a chaperone released by substances inside the tumor. The vaccine currently targets patients with advanced metastatic colorectal cancer. It can be used as a tumor antigen and adjuvant to regulate the immune response *in vivo*. The aim of the CryoVax vaccine is to create “hot” tumors in these patients and then naturally block the expression of checkpoint molecules. Currently, AlloVax vaccine is mainly used in patients with advanced liver cancer. The chaperones in tumor cells carry autologous tumor-specific peptides (antigens) that confer tumor-specific immunity. AlloVax vaccines contain protein-associated cell lysates (CRCL). After AlloVax injection, CRCL contains a lot of tumor antigens, so it increases the chances of the body producing an effective immune response to all tumors. Ronald Levy and other researchers developed a new cancer vaccine, irradiation of iPSCs as an autogenous anti-tumor vaccine. They found that the new cancer vaccine is suitable for many different types of cancer, including breast cancer and colorectal cancer (27).

### CAR-T Cell Immunotherapy

**Figure 2:**
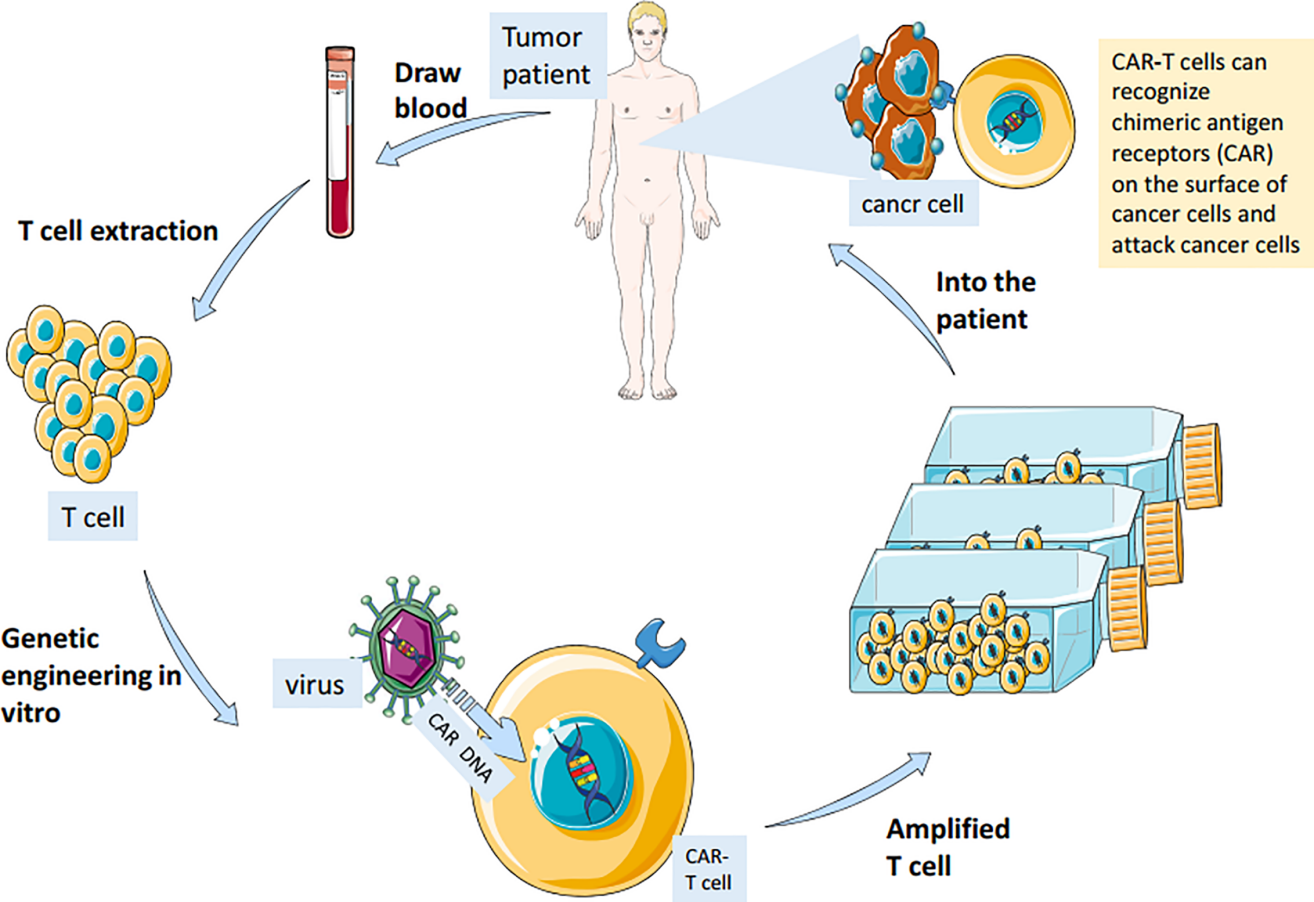
Immune T cells were isolated from patients and genetically engineered *in vitro* to be fitted with chimeric antigen receptors (CAR) that recognize cancer cell surface antigens. The modified cells are amplified in large Numbers *in vitro* and injected back into the patient to achieve the therapeutic effect of accurately identifying and killing cancer cells.

CAR-T cell immunotherapy (chimeric antigen receptor T cell immunotherapy) is a new type of cancer immunotherapy. Car-T therapy extracts some T cells from patients and gene modification them to make T cells express new receptor CAR. After proliferation, they are infused back into the patient’s body (**Figure 2**). These T cells can quickly identify and destroy target cells using their CAR receptors (28). Compared with surgery, radiotherapy, chemotherapy, targeted therapy and hematopoietic stem cell transplantation, CAR-T cell immunotherapy is more accurate, flexible, spectral and durable. It has a remarkable curative effect in the treatment of acute leukemia and non-Hodgkin lymphoma (29). In a recent study, researchers found that CAR-T therapy is effective in treating patients with glioblastoma and can remove 80% of the tumors (30). Moreover, the combination of anti-cancer vaccine and CAR-T therapy can stimulate the immune system to produce memory T cells and prevent the recurrence of tumors (31, 32).

### TCR-T Therapy

Although the existing CAR T treatment has shown significant efficacy in clinical trials for acute and chronic lymphoblastic leukemia, the available targets for CAR T treatment are limited, the treatment of solid tumors has not been very effective, and the adverse reactions caused by CAR T treatment are sometimes difficult to control. Compared with CAR T, TCR-T therapy can select more targets and has better efficacy in solid tumors with fewer side effects. TCR-T therapy improves the affinity and combat effectiveness of TCR (T cell antigen receptor) that specifically recognizes tumor-associated antigen by transducing chimeric antigen receptor or TCR α/β heterodimer, enabling T lymphocytes to re-efficiently recognize target cells (33).

In a collaborative trial study, researchers found that preliminary clinical results from patients receiving TCR-T cell therapy showed encouraging positive signs. TCR had better binding affinity after improvement. TCR-T cells showed excellent expression level. The persistence of therapeutic effects has been demonstrated in preliminary studies. In addition, researchers have developed other HLA subtypes to treat more patients with different HLA subtypes in the future. At present, more and more enterprises at home and abroad have carried out research on TCR-T therapy.

### Fusion Cell Therapy

Fusion cell therapy is a kind of therapy that uses cancer cells of patients to develop new dendritic cells to attack cancer cells. Through the direct fusion of cancer cells and dendritic cells of cancer patients, new dendritic cells are cultivated. When the new dendritic cells are reinjected near the lymph nodes, they will educate T cells that can remember a lot of cancer antigen features. Even if the cancer cells hide a feature, the T cells will recognize them from other features, leaving the cancer cells nowhere to hide, and finally be killed. A phase II trial of fused cell vaccine + IL-12 in 15 patients with brain tumors (gliomas) showed that the treatment prevented 73 percent of the disease from deteriorating, with a clinical response rate of 40 percent (34). Avigan et al. has investigated the efficacy of the fused cell vaccine in treating kidney cancer (35), showing that the vaccine contains both dendritic cells of the patient and the patient’s own cancer antigen, which can induce a wide immune response and make it difficult for cancer cells to escape under the surveillance of the immune system. Avigan et al. found that the combination of the fused cell vaccine and anti-PD-1 antibodies was also applicable to blood cancers such as leukemia and myeloma (36). Because the vaccine is based on the patient’s cells, a completely customized cancer vaccine modulation is realized without any side effects.

### CAR - NKT Therapy

The researchers found that natural killer T cells (NKT) are a special subset of T cells that have both the T cell receptor TCR and the NK cell receptor on their surface (37). NKT cells destroy cancer cells by releasing cytotoxic particles (38). Car-nkt cell therapy is to separate the NKT cells from the blood of patients or healthy people, and collect them back to the patients after reaching a certain amount of culture *in vitro*. Researchers conducted a phase I clinical trial based on engineered CAR-NKT cells to evaluate CAR-NKT cell therapy in children with recurrent neuroblastoma. At present, two children with neuroblastoma have been treated. One patient was stable at 4 weeks follow-up. The other patient had two bone tumors, one of which disappeared completely (39).

### LN - 145 Therapy

LN-145 is a therapy (40) developed by Iovance which amplifies and activates the anti-tumor activity of autologous TILs *in vitro*, and returns them to the patient. TIL is a special lymphocyte mobilized by the immune system in the early stage of cancer. These lymphocytes have the ability to recognize and attack the flow of tumors and penetrate into tumors. LN-145 therapy extracts TIL from patients and then stimulates TIL amplification *in vitro* with IL-2 cytokines. This not only increases the number of TIL, but also activates TIL’s anti-tumor ability. Finally, these TILs were injected back into the patient to play a more powerful role in killing cancer cells. LN-145 therapy has shown amazing data in clinical trials (41). FDA approved LN-145 for breach-through treatment of advanced cervical cancer and accelerated its approval for listing.

### Checkpoint Inhibitor

After the activation of T cells involved in anti-tumor immune response, the expression of various inhibitory regulatory receptors on the surface of T cells was up-regulated, and combined with the corresponding ligands highly expressed on the surface of tumor cells, which inhibited the immune response and down regulated the intensity of tumor related immune response. In the process of immune response, the sites with inhibitory immunoregulation are called immune checkpoints. Traditional immunotherapy is mainly to induce or strengthen anti-tumor immune response, but due to the existence of immunosuppressive immune regulation, such as immune checkpoints, often cannot produce lasting and effective anti-tumor immune effect. Checkpoint inhibitors can effectively block the inhibitory immunoregulation of PD-1/PD-L1, CTLA-4 and other immune checkpoints, so as to indirectly strengthen the anti-tumor immune response and improve the effect of immunotherapy. The combination of these two checkpoint inhibitors has also been evaluated clinically for improved efficacy but increased toxicity. The anti-CTLA-4 antibody ipilimumab was shown to be effective for advanced melanoma for the first time (42–44). At present, CTLA-4 and PD-1 inhibitors have been used in clinical immunosuppression. Among them, CTLA-4 inhibitors include apilimumab, tremelimumab, etc. PD-1 inhibitors include nivolumab, pembrolizumab, pidilizumab, etc. In recent years, the biomarkers of immunocheckpoint inhibitors have developed rapidly. This is due to the research on the mechanism of the interaction between tumor and host genome, tumor microenvironment and immune function. T cells are activated by two signals (TCR-MHC-peptide recognition antigen and CD28 something CD 80/86 costimulatory signal). Mutations in Tumor Cell Genomes or proteins that are abnormally expressed are processed into peptides that bind (or fail to bind) to MHC class I molecules. Immunogenic peptides (MHC-bond immunogenic peptides) trigger a subsequent immune response. Generally, the higher the mutation load, the higher the possibility of producing MHC-bond peptides. Inhibitory immune cells (such as tumor-associated macrophages) and tumor-causing inflammatory mediators (such as TNF-α、 IL-6、 TGF-β) in tumor microenvironment can stimulate tumor cell proliferation and induce tumor angiogenesis by inhibiting NF-κB and STAT3 signaling pathways, promote the tumor immune escape as well as the tumor invasion and metastasis (45–47).

### Regulatory T Cells

Tregs is a subtype of CD4 cells, which can inhibit immune response, maintain immune tolerance and prevent autoimmune. Tregs can enhance immune function and help maintain homeostasis. In cancer, however, Tregs infiltrate the tumor microenvironment (7, 48, 49). This is associated with poor prognosis and poor response to immunotherapy. In animal models, the removal of Tregs has been shown to improve the anti-tumor immune response. CTLA-4 is highly expressed on Tregs. Treatment with anti-CTLA-4 antibody could deplete Tregs in tumor microenvironment in mice. This is achieved through a mechanism that relies on tissue to host macrophages. However, some studies have shown that enhanced tumor therapy *via* anti-CTLA-4 antibody is dependent on interactions with Tregs and T effectors (50, 51).

## PD-1/PD-L1 and Tumor Immunotherapy

In normal and stable state, PD-L1 pathway can maintain immune homeostasis and protect immune system. In canceration, PD-L1 can disrupt the tumor’s immune cycle in two ways, thus protecting the tumor from damage by cytotoxic T cells. First, over-expression of PD-L1 on the surface of tumor-infiltrating immune cells in lymph nodes prevents the initiation and activation of new cytotoxic T cells and their recruitment into the tumor (52). Secondly, in the tumor microenvironment, the up-regulation of PD-L1 on the surface of dendritic cells leads to the inactivation of cytotoxic t cells. In both cases, PD-L1 interacts with PD-1, a homologous ligand on the surface of T cells, which can inhibit the function of T cells, alter their phenotypes, induce T cell tolerance, inhibit T cell proliferation, and reduce cytokine production, blocking T cell recognition of tumor cells (53, 54).

### Autophagy

Tumors often adapt to resource deprivation through different survival mechanisms, such as autophagy. Autophagy is the process of degradation and recycling of self-nutrients through lysosomal pathway, clearing away damaged organelles and protein aggregates, thereby maintaining cell steady-state catabolism. Autophagy is beneficial to normal cells, but in tumors, it helps malignant cells adjust and adapt to adverse conditions, allowing them to develop and continue to grow. In addition, the PD-1/PD-L1 signaling pathway plays a key role in tumor function and survival (55, 56). Autophagy is influenced by the PD-L1 ligand. The results of mouse melanoma cells and human ovarian cells showed that cells with high expression of PD-L1 receptor were more sensitive to autophagic inhibitors than cells with low expression of PD-L1 (57). Preclinical studies in mice with human melanoma xenografts have shown that the combination of Anthracycline and autophagy inhibitors enhances the anti-tumor T cell response. The same study also showed that autophagy inhibitors have no significant side effects on the immune system. These results provide new opportunities for cancer therapy, such as drugs targeting the PD-1/PD-L1 axis in combination with autophagy inhibitors. Although autophagy inhibitors in combination with anti-PD-L1 agents offer a new direction for cancer therapy, autophagy inhibitors are only one part of a complex network of pathways that affect the immune system’s role in tumor cell death, further research is needed to identify the various scenarios in which autophagy inhibitors are effective against cancer (58).

### Tumor-Associated Macrophages

A study has shown that both rats and humans express PD-1 in their Tumor-associated macrophage. As the tumors proliferated, the expression of PD-1 increased significantly in both mice and human tumor-associated macrophage. The higher the expression of PD-1 in tumor-associated macrophage, the lower the phagocytosis of tumor cells. Blocking PD-1/PD-L1 *in Vivo* can increase the phagocytosis of macrophages, slow down the growth of tumor and prolong the survival time of tumor model in mice. The greater the number of megakaryocytes given, the longer the survival time. Thus, PD-1/PD-L1 therapy can also play a role through macrophages, which has substantial significance for the creation of new tumor immunotherapy methods (59, 60).

### CD47

CD47 is a widely expressed transmembrane glycoprotein, also known as integrin-associated Protein (IAP), an immunoglobulin superfamily protein that is expressed on the surface of almost every cell in the body. CD47 protects healthy cells from the immune system by binding to the n terminal of the immune cell signal-regulating protein α (SIRPα), signaling “don’t eat me” and inhibiting the phagocytosis of macrophages. In the meantime, CD47, as a “don’t eat me” signal, protects tumor cells from phagocytosis by macrophages, which has become a new mechanism of tumor development and development, and has also explored a new effective way for tumor immunotherapy (61, 62). CD47 antibodies target a variety of indications, including hematological malignancies such as non-Hodgkin Lymphoma and acute myeloid leukemia (AML), as well as solid tumors such as colorectal cancer, ovarian, and bladder cancers (63).

## Screening and Evaluation of the Benefit Group Before Immunotherapy

Due to the remarkable efficacy of immunotherapy in tumor therapy, immunotherapy is regarded as one of the most promising therapeutic strategies in the field of tumor therapy in recent years. Immuno-checkpoint inhibitors have brought great changes to the treatment model of advanced tumors, but the clinical treatment results show great individual differences (64). Only a small number of patients show a lasting response to the drug, most patients do not show a lasting response. It has become an especially urgent problem for doctors and patients how to predict the curative effect and screen the effective immunotherapy population before treatment.

### Tumor Microenvironment Scoring System

Zeng et al. found that the tumor microenvironment scoring system is not only related to the prognosis of tumor patients, but also has important value for the screening of immunotherapy beneficiaries. Through PCA algorithm, they scored the tumor microenvironment of gastric cancer patients, and found that the higher the tumor microenvironment score, the stronger the anti-tumor immune response-ability, the greater the potential of benefiting from immunotherapy, and the longer the survival time of gastric cancer patients (65).

### TIDE Algorithm

Studies have shown that T-cell function-related tumor immune dysfunction and rejection (TIDE) algorithm can predict the efficacy of immunotherapy. TIDE score was high, the efficacy of immune checkpoint blocking therapy (ICB) was poor, and the survival period after ICB treatment was short. TIDE is better than other markers such as PD-L1, mutation load and IFN-γ in predicting the efficacy of first-line treatment of melanoma with ICB (66).

### PD-1/PD-L1

Initial studies focused on the expression of PD-1/PD-L1 in tumor cells and surrounding immune cells. A team found that PD-1/PD-L1 antibodies work by mobilizing macrophages infiltrating tumor tissue to gobble up and destroy tumor cells (58, 67, 68). It is believed that the higher the expression of PD-1/PD-L1, the better the effect of immunotherapy. It does present this trend in clinical practice. However, some patients with negative expression of PD-1/PD-L1 also benefit from the treatment of PD-1/PD-L1 monoclonal antibody (59, 69), so it is not recognized as the only precise biomarker to guide immunotherapy.

### Tumor Mutation Load

Through total exon sequencing, Yang et al. found that tumor patients with good therapeutic benefits generally have high somatic mutation load. At the same time, patients with high tumor mutation load are more likely to benefit from anti-CTLA-4 treatment (70). The researchers found a similar pattern in non-small cell lung cancer. In general, the higher the mutation load of the tumor, the stronger the immune response provoked, and the better the effect of immunotherapy. In addition, microsatellite instability (MSI) (21) and DNA mismatch repair functional defect (dMMR) are also potential biomarkers. In the analysis of more than 60,000 tumor samples, Volkov et al. found that MSI-H patients generally had high tumor mutation load (71). On the other hand, it also reflects that the tumor mutation load is correlated with the benefit of immune checkpoint inhibitors (72, 73). Tumor patients with MSI-h characteristics, or tumor patients with mismatched repair gene defect (dMMR), tend to benefit from immune checkpoint inhibitor therapy (74). FDA announces MSI as a molecular diagnostic marker for tumor immune checkpoint inhibitor therapy. This is great progress in using standard biomarkers to guide immunotherapy.

### Classical Monocytes With CD14+CD16-HLA-DRhi Phenotype

Researchers selected 20 melanoma patients as study subjects (75) and found that the proportion of classical monocytes with CD14+CD16-HLA-DRhi phenotype in the peripheral blood of patients can be used as biomarkers for predicting PD-1 drug reactivity. According to the experimental model, the researchers determined 19.38% as the optimal threshold, that is, when the subgroup ratio of CD14+CD16-HLA-DRhi is greater than 19.38%, the treatment regimen with antibody PD-1 is recommended for patients. The results show a bright application prospect of this research.

### Invasive T Cells and T Cell Receptor

Tumors with more invasive T cells (CD8+, killer T cells) surrounding cancer cells are known as “hot” tumors. Numerous studies have found that immune checkpoint inhibitors have a very good effect on “hot” tumors (76, 77). Moreover, it is theoretically believed that the more extensive the expression spectrum of T cells after treatment, the more it can reflect that the drug activates the immune function of the body. In fact, some studies have found that immune checkpoint inhibitors have limited effects on “hot” tumors. Scheper et al. argues that rather than categorizing a tumor as “hot” or “cold” simply by the number of T cells it has infiltrated, it should be determined whether the TCR of these t-cells can recognize a tumor (78). They set up a platform to analyze whether infiltrating T cells in tumors can recognize surrounding cancer cells. TCR can be used to accurately analyze whether killer T cells in tumors have the potential to fight cancer. Schumacher et al. (79) suggest that increasing the coverage of TCR on the surface of T cells infiltrating tumors may enhance the therapeutic effect of immune checkpoint inhibitors.

### CD39

Simoni et al. found that CD39 may be a predictive biomarker for patients to respond to CD8 + TIL targeted cancer immunotherapy. Whether CD8+ TIL cells express CD39 or can predict the response degree of patients to PD-1 antibody therapy, it is of great help to screen more potential anticancer T cells in TIL and CAR-T cell therapy, and further expand the immunotherapy (80–82).

## Conclusions, Problems and Prospective

Immunotherapy of tumor is a very popular anti-tumor treatment method in recent years, and is expected to become the main means of anti-tumor treatment in the future. Based on large number of research results, it is not difficult to find that tumor immunotherapy has shown amazing clinical efficacy in many types of cancer, such as melanoma, lung cancer, and cancer with high mutation load of microsatellite instability. Immunotherapeutic drugs have shown excellent results, whether used alone or in combination with other therapies. The rapid progress of PD-1 inhibitors and PD-L1 inhibitors is regarded as a star product to open a new era of cancer immunotherapy. With the discovery of new targets for immunotherapy and the development of new drugs for immunotherapy, people’s confidence in overcoming cancer has become more and more firm.

Fortunately, more and more studies have found breakthroughs to improve the efficacy of immunotherapy and reduce side effects, such as thioredoxin, FATP2, bacterial transplantation, etc. Moreover, effective indicators such as tumor mutation load index and tumor microenvironment scoring system can be used to screen the population that can benefit from immunotherapy and evaluate the efficacy. Believe it’s only a matter of time before cancer is conquered (83). Through the unremitting efforts of human beings, we can certainly rekindle the hope of life for cancer patients earlier.

## Author Contributions

XZ conceived and designed the study. ZW and SL wrote this manuscript. ZW, SL, and XZ discussed and edited the manuscript. All authors contributed to the article and approved the submitted version.

## Funding

This work was supported partly by National Natural Science Foundation of China (81541153), Guangdong Key Laboratory funds of Systems Biology and Synthetic Biology for Urogenital Tumors (2017B030301015) and Doctoral Research Initiation Fund of Guangdong Medical University (B2012001). The funders had no role in study design, data collection and analysis, decision to publish, or preparation of the manuscript.

## Conflict of Interest

The authors declare that the research was conducted in the absence of any commercial or financial relationships that could be construed as a potential conflict of interest.
